# Ross Ice Shelf frontal zone subjected to increasing melting by ocean surface waters

**DOI:** 10.1126/sciadv.ado6429

**Published:** 2024-11-08

**Authors:** Peter M. F. Sheehan, Karen J. Heywood

**Affiliations:** Centre of Oceanic and Atmospheric Sciences, School of Environmental Sciences, University of East Anglia, Norwich, UK.

## Abstract

Solar-warmed surface waters subduct beneath Antarctica’s ice shelves as a result of wind forcing, but this process is poorly observed and its interannual variability is yet to be assessed. We observe a 50-meter-thick intrusion of warm surface water immediately beneath the Ross Ice Shelf. Temperature in the uppermost 5 meters decreases toward the ice base in near-perfect agreement with an exponential fit, consistent with the loss of heat to the overlying ice. Ekman forcing drives a heat transport into the cavity sufficient to contribute considerably to near-front melting; this transport has increased over the past four decades, driven by the increasing heat content of the ice-front polynya. Interannual variability of the heat transport is driven by zonal wind stress. These results provide a benchmark against which model performance may be assessed as we seek to reduce uncertainty around the contribution of basal melting to sea level rise.

## Teaser

The heat advected beneath the Ross Ice Shelf by surface water, observed here directly, has increased over the past 40 years.

## INTRODUCTION

The ice shelves that surround Antarctica are exposed to the warmth of the ocean across the expanse of their undersides that float out over the continent’s shelf seas, and the ocean-driven melting that occurs at the ice base is the largest cause of Antarctic ice-mass loss (*1*–*3*). Although the melting of floating ice does not itself substantially raise the sea level, ice shelves slow the seaward flow of land ice and so stabilize the Antarctic ice sheet; their thinning and disintegration would hasten the delivery of land ice to the ocean and accelerate global sea level rise (*4*). Moreover, even spatially and temporally localized melting can influence ice flow far upstream, up to, and including, grounded tributary glaciers (*5*).

Much research to date has focused on understanding how warm Circumpolar Deep Water (CDW) from the Antarctic Circumpolar Current is brought onto the continental shelf and directed toward ice shelf cavities. Commonly found at the right depth to access these cavities (below ~300 m), CDW is the principal driver of basal melting of ice shelves around many of Antarctica’s shelf seas (*6*, *7*). However, the Ross Sea experiences little intrusion of CDW: Bottom temperatures are generally between −1.5° and −2°C (*8*). Consequently, and unlike the fast-melting ice shelves of West Antarctica, the average basal melt rate of the Ross Ice Shelf, which comprises about a third of Antarctica’s total ice shelf area, is very low (0 to 0.3 m year^−1^) (*1*, *2*) and temporally stable (*9*), although rates are higher (up to 8 m year^−1^) in the immediate vicinity of the ice front (*10*, *11*). Nevertheless, surface waters, warmed during summer to ~0.5°C and driven into cavities by, for instance, surface wind stress, can provide an additional source of heat to drive basal melting (*9*, *11*–*16*), and given that the thin layer of water immediately beneath the Ross Ice Shelf is very close (within 0.02°C) to the in situ freezing temperature (*17*, *18*), the melt rate of the Ross Ice Shelf could be sensitive to even relatively small-scale intrusions that elevate the temperature of this under-ice boundary layer.

These surface-water intrusions have not yet been observed directly as they enter an ice shelf cavity: Moorings are not routinely deployed close to an ice front, and autonomous underwater vehicles do not venture close enough to the ice base (i.e., 10 to 20 m) to sample any surface-water layer (*19*–*22*); any vehicle with a propeller would likely disturb the boundary layer such that its observations would be meaningless. Present understanding of surface-water intrusions is consequently derived principally from comparisons of hydrography in open water, perhaps from ship (*11*) or seal (*14*) observations, and from through-ice moorings deployed well within a cavity (*11*). Such observational constraints also mean that the upper-cavity boundary layer, immediately beneath the ice base and of crucial importance for understanding the transfer of heat from ocean to ice, is underobserved. The long-term (i.e., interannual to decadal) variability of surface-water intrusions, including how they might have responded to climate change, is yet to be investigated.

Here, we present observations collected by an ocean glider that accidentally sampled the cavity beneath the Ross Ice Shelf to a depth of 200 m. The observations reveal an intrusion of warm surface waters from the adjacent Ross Sea polynya immediately beneath the ice, which we use to characterize the under-ice boundary layer. We then aim at placing surface-water intrusions into a climatic-scale context on a shelf-wide scale. We calculate the Ekman heat transport across the front and demonstrate that the heat advected into the Ross Ice Shelf cavity by wind-forced surface-water intrusions has increased over the past four decades.

## RESULTS

### Direct observations of a warm, surface-water intrusion

**Fig. 1.**
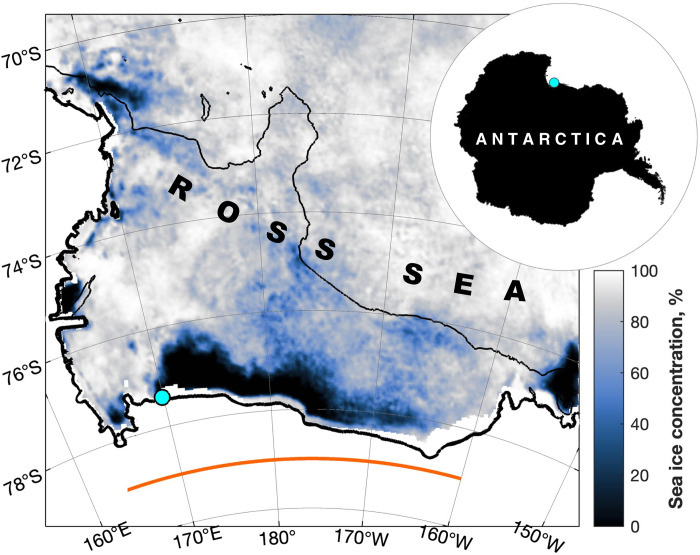
Mean sea ice concentration while the Seaglider was under the ice, i.e., 4 to 8 December 2022. The glider was deployed into the polynya from fast ice at the location indicated by the bright blue circle; the deployment location is similarly indicated on the inset (top left). The thick black line indicates the ice shelf edge, taken from BedMachine v3; the thin black line indicates the 1000-m isobath. The orange line indicates the longitudes included in along-front integrals and averages.

A Seaglider (SG613) was deployed in the southern Ross Sea on 4 December 2022 from the edge of the shelf-fast sea ice, only some 100 m from the front of the Ross Ice Shelf (Fig. 1). A Seaglider (*23*) is an autonomous underwater vehicle that collects V-shaped, depth-distance profiles of ocean properties. To conserve power in the interests of endurance, Seagliders lack a propeller or any similarly power-hungry engine: Instead, they modify their buoyancy by either filling or draining a shielded external bladder housed in the rear of the external casing. Rear-fitted fins convert some of the ensuing vertical motion into horizontal motion; rotating the asymmetrically weighted battery enables steering. Maneuverability is limited and navigation is crude, and gliders struggle to make headway against strong currents. Gliders communicate using the GPS satellite network, but only when at the surface; underwater, they navigate by dead reckoning alone. A glider that “surfaces” under an obstacle such as sea ice or an ice sheet will not be able to determine its location. In this situation, it will execute a subsequent dive in a preprogrammed direction, continuing until it next surfaces in open water and manages to communicate successfully. The mean time between the final observation of a given dive and the first observation of the subsequent dive (during which period the glider was attempting to make contact via satellite) was 25 ± 3 min.

**Fig. 2.**
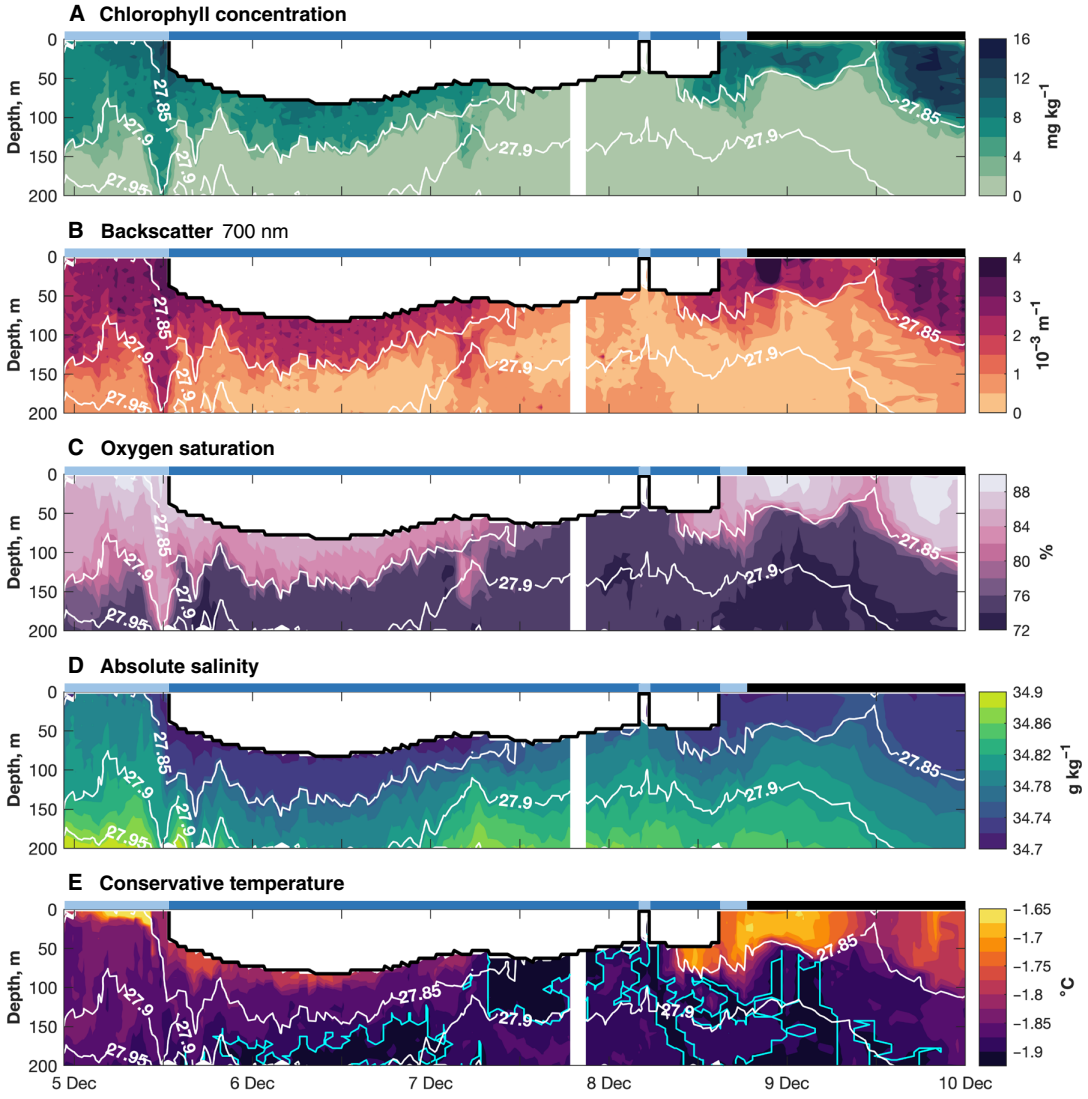
Time series of hydrographic observations collected by SG613. (**A**) Chlorophyll concentration (mg kg^−1^), (**B**) particulate backscatter intensity (m^−1^), (**C**) oxygen saturation (%), (**D**) absolute salinity (g kg^−1^), and (**E**) conservative temperature (°C), with supercooled water enclosed within the bright blue lines. In (A) to (E), white contours indicate potential density (kg m^−3^); light blue top bars indicate profiles under sea ice, dark blue top bars indicate profiles under the ice shelf, and black top bars indicate profiles in open water.

A trip into the cavity underneath the Ross Ice Shelf was not planned. Before deployment, we had programmed the glider to travel immediately north and begin a sampling campaign in the Ross Sea polynya. However, after deployment, the glider did not communicate again until 8 December, having completed 79 dives. The uppermost observation on many of these dives was between 40 and 80 m below sea level (Fig. 2), indicating a “surfacing” under the Ross Ice Shelf; this interpretation of deep surfacings in the vicinity of an ice shelf follows that of Nelson *et al.* (*17*), who collected 30 dives beneath the Ross Ice Shelf in 2010. The shallow draft of the ice base suggests that the glider observed beneath the thinnest, near-front region of the ice shelf. Dives on which the uppermost observation was ~1 m below sea level indicate a surfacing under sea ice.

While navigating by dead reckoning and unable to determine its overground velocity, the glider nevertheless recorded an estimate of its through-water velocity, which was consistently northward. Using a tide model (*24*, *25*), we estimate that the region’s diurnal tidal currents alone would have taken the glider about 2 km south of its deployment location (see the Supplementary Materials); although because the glider did not return to this location after a diurnal cycle and because it was actively moving relative to the water, we doubt that tidal currents alone are responsible for the accidental foray beneath the ice. Summertime background currents in the region, as observed by under-ice moorings, are on the order of a few centimeters per second and are directed to the south or southeast (*11*); this is clearly consistent with the glider being advected southward beneath the ice shelf. Unfortunately, given the helicopter-supported nature of the deployment, ship acoustic Doppler current profiler observations are not available. Nevertheless, given the magnitude of the expected background flow, we estimate that the glider traveled between 1 and 5 km southward under the ice from its deployment location. More details on our estimate of the glider’s under-ice penetration are provided in the Supplementary Materials.

We observe a 50-m-thick intrusion of warm surface water immediately beneath the Ross Ice Shelf, from just after midday universal time (UT) on 5 December to around 09:00 UT on 7 December (Fig. 2; see Materials and Methods). The clearest indication of the near-surface, open-ocean origin of this under-ice water is its high-chlorophyll concentration (Fig. 2A), which cannot have developed in situ: Conditions under an ice shelf are too dark to support photosynthesis and phytoplankton growth. We also note that the glider being advected into the cavity from the polynya, despite its attempts to travel northward, suggests an open-ocean origin for the observed waters. This high-chlorophyll surface water is also turbid, oxygenated, warm, and fresh (Fig. 2, B to D). The lower boundary of this surface-water intrusion is the 27.85 kg m^−3^ isopycnal (Fig. 2). However, under the sea ice, prior to the glider’s foray into the cavity proper, the high-chlorophyll, high-turbidity, high-oxygen water extends down to the 27.9 kg m^−3^ isopycnal; this suggests that flow into the cavity occurs only for waters lighter than 27.85 kg m^−3^. We note that gradients between water masses are sharp.

Within this surface-water intrusion, the warmest temperatures (>−1.8°C) are found in the uppermost cavity. Colder water, much of it potentially supercooled—i.e., colder than the surface-pressure freezing temperature (*26*, *27*)—is observed immediately beneath the ice base on 7 and 8 December; this water is denser than 27.85 kg m^−3^, which intersects the ice base at approximately 12:00 UT on 7 December. Before lastly exiting the cavity, the glider again observes warm, fresh surface waters in contact with the ice base (Fig. 2A). Given that we have no information on the glider’s trajectory beneath the ice shelf, we cannot estimate the horizontal extent of these different water masses and thus of the surface-water intrusion. Nevertheless, given that the glider emerged into the polynya only 6 km from where it was deployed, it seems likely that the length scale of the intrusion is relatively small (i.e., order a few kilometers).

In the immediate vicinity of the front, both under sea ice and in open water, the upper ocean is variable in time. The glider observes a sharp descent of the 27.85 kg m^−3^ isopycnal as it first crosses the ice Ross Ice Shelf front into the cavity on 5 December (Fig. 2). This horizontal, cross-front gradient in density is consistent with an along-ice-front, westward-flowing current, perhaps similar to the Antarctic Coastal Current observed in the Bellingshausen and Amundsen Seas (*28*)—although this would appear to be closer to the ice front and on a much smaller scale than in either of those two basins (*28*). This same feature is not present as the glider lastly exits the cavity on 8 December (Fig. 2). Also, the near-surface layer observed as the glider exits the cavity is lighter than 4 days before (<27.85 kg m^−3^), and the high-chlorophyll, high-turbidity, high-oxygen water extends down only to the 27.85 kg m^−3^ isopycnal, rather than to the 27.9 kg m^−3^ isopycnal (Fig. 2, A to C). An along-ice-front current might present a dynamic barrier to transport into and out of the cavity, modulating the intrusion of warm surface waters and the export of, for instance, meltwater plumes. Not having information on the glider’s position beneath the ice, we cannot estimate velocities from the present observations; however, future ocean observations in any similar region that can be used to estimate velocity could help characterize any such feature.

### The under-ice boundary layer

**Fig. 3.**
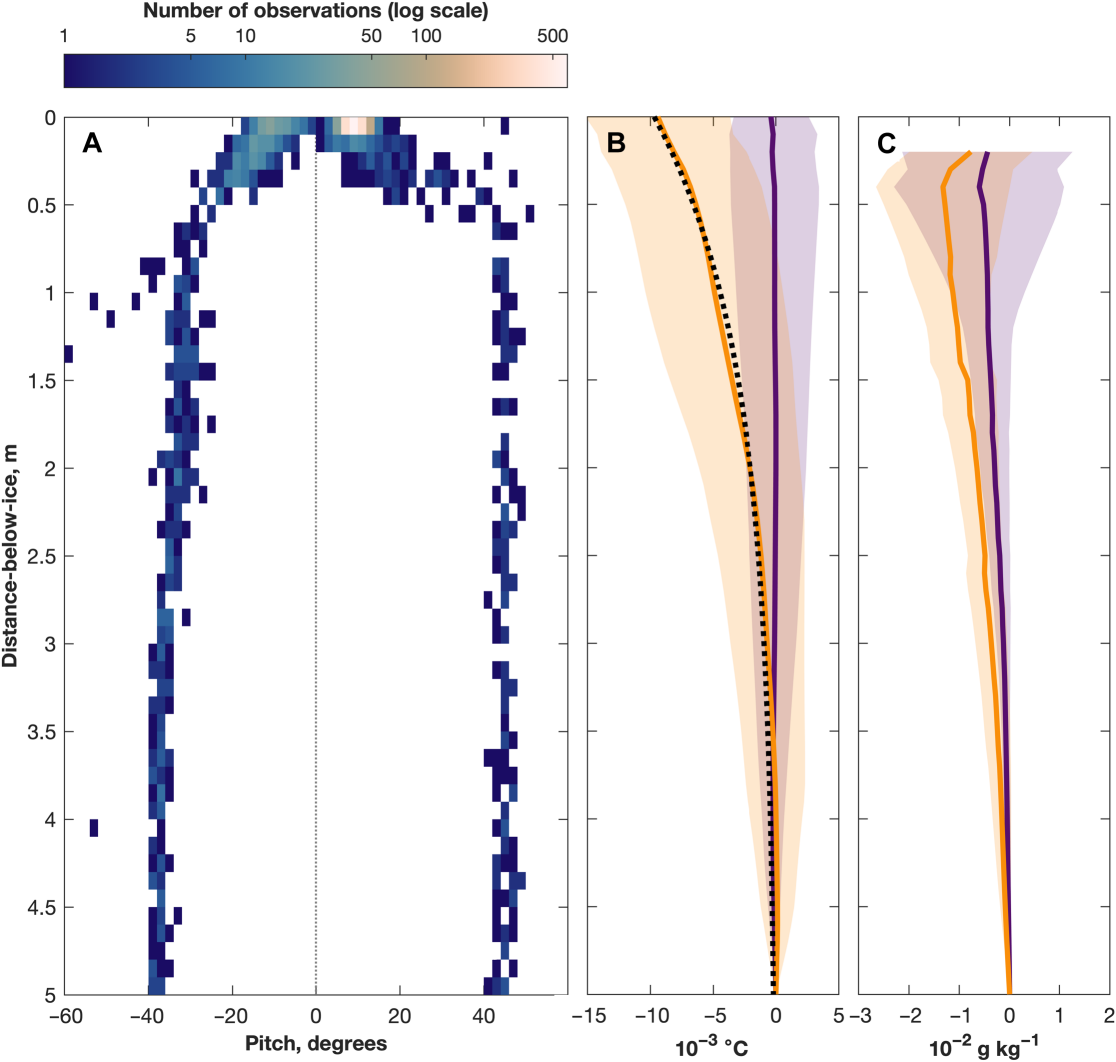
The under-ice boundary layer. (**A**) Histogram of the glider’s pitch (degrees; i.e., the angle that the longitudinal axis makes with the horizontal) against distance-below-ice (m). (**B**) Mean relative profiles (thick lines) of conservative temperature (°C) plotted against distance-below-ice for warm-water profiles (orange) and cold-water profiles (purple). The shaded regions enclose ±1 SD, and the black dotted line is the exponential fit to the mean relative warm-water profile. (**C**) As in (B), but for absolute salinity (g kg^−1^).

The glider observations allow us to characterize in detail the boundary layer that is the top few meters beneath the ice shelf base, particularly because of the high density of observations that the glider collected there. Given that the glider did not expect to encounter the base of the Ross Ice Shelf, it remained positively buoyant, sufficiently so to rise to the ocean surface, even as its upward motion was arrested by the ice. Consequently, the glider’s pitch—i.e., its angular offset from the horizontal—decreased sharply in the upper 1 m or so of the water column as the still-buoyant glider flattened itself against the ice base (Fig. 3A); science sensors remained sampling during this time. This resulted in a high density of observations from immediately beneath the ice base (Fig. 3A); the unusually low pitch of the glider was such that the distance between the temperature and salinity sensors, both located on the upper part of the glider’s outer fairing, and the ice base was minimal.

We now exploit the observations from beneath the ice shelf to analyze the under-ice boundary layer. (We exclude dives from beneath the sea ice from this analysis.) We consider *T_r_*(*z*′): profiles of conservative temperature against distance-below-ice, *z*′, relative to the temperature 5 m below the ice, *T*(5)

$$T_{r}\left(z\prime \right)=T\left(z\prime \right)-T\left(5\right)$$ (1)

Furthermore, we separate the under-ice profiles into those where warm water is in contact with the ice base (i.e., within the surface-water intrusion) and those where cold water is in contact with the ice base (see Materials and Methods). We perform the same calculation with absolute salinity.

In the mean cold-water profile, temperature in the uppermost 5 m is nearly constant with distance-below-ice (Fig. 3B). In contrast, in the mean warm-water profile, the temperature in the uppermost 5 m decreases exponentially toward the ice base (Fig. 3B). The mean warm-water profile is described by the curve

$$T_{r}\left(z\prime \right)=-0.0097e^{-0.77z^{\prime }}$$ (2)

The agreement between the mean warm-water profile and Eq. 2 is very good (*R*^2^ = 0.99). The lack of a well-defined mixed layer or any step-like temperature structures is in contrast to profiles collected close to the grounding line of the Ross Ice Shelf, which exhibit a subglacial mixed layer of ~1 m in thickness immediately beneath the ice (*29*). Furthermore, we suggest that the lack of an under-ice mixed layer is because turbulent mixing, such as might lead to a scalloped ice base (*22*), is low, at least during the period of our observations.

The decrease in temperature toward the ice base must indicate heat loss from the upper water column to the overlying ice and perhaps the addition of meltwater. No such heat loss occurs from cold water: Being at or below the in situ freezing point, the cold water has little to no heat to lose. If we assume that the relative cold-water profile is representative of a situation without heat loss to the ice, then we may integrate the difference between the warm-water and cold-water profiles (see Materials and Methods); thus, we determine that the heat lost to the overlying ice from the warm surface-water intrusion is 4.74 × 10^4^ J. Given that we cannot determine how long the observed surface-water intrusion has been under the Ross Ice Shelf, we lack information on the timescale over which this heat was lost and so cannot calculate a flux. However, if we assume timescales, then we can estimate the following: if the heat were lost over a period of a day, the flux would be 0.55 W m^−2^; if the heat were lost over a period of a week, the flux would be 0.08 W m^−2^. Average salinity in the uppermost 5 m is lower in warm-water profiles than in cold-water profiles (Fig. 3C), although the relatively large SDs suggest that the difference between warm-water and cold-water salinity profiles is less marked than between warm-water and cold-water temperature profiles.

We have presented high-resolution observations of the oceanic boundary layer at the top of an ice shelf cavity, close to the ice shelf front. We observe warm water in contact with the ice base; the similarity between the physical and biogeochemical characteristics of the intrusion and the near-surface waters of the adjacent ice-front polynya suggests a recent, open-water origin for the intrusion. Within the warm-water intrusion, the temperature in the top 5 m of the cavity decreases exponentially with distance toward the ice base as the ocean surrenders its heat to the overlying ice shelf. Outside of this intrusion, where temperatures in the upper cavity are at or very close to the in situ freezing point, no such temperature decrease is observed, the ocean having almost no heat to surrender. These results provide observational confirmation of results that have hereto been based only on theory and modeling, and they present a benchmark against which model performance may be assessed. Next, we consider the implications of these findings and extrapolate them temporally to investigate long-term change.

### Interannual variability of wind-forced surface-water intrusions

The above results present a snapshot of ice-ocean interaction: a crucial interface of the global climate system but one that is extraordinarily challenging to observe directly. Given the scarcity of previous observations, the influence of surface-water intrusions on climate-relevant timescales is yet to be investigated. Hence, we now embark on a broader consideration of intrusions into the Ross Ice Shelf cavity to further our understanding of the context and wider climatic significance of intrusions such as we observed. Surface-water intrusions may be driven by various processes and have been identified in observations (*11*) and models (*15*, *30*, *31*). In particular, we note that surface Ekman currents (*14*) and density-driven currents (*11*, *30*) have been found to promote incursions into the Ross Ice Shelf cavity. Here, we do not aim at a comprehensive assessment of all processes that might drive surface-water intrusions into the cavity. Rather, we focus on surface Ekman flows.

**Fig. 4.**
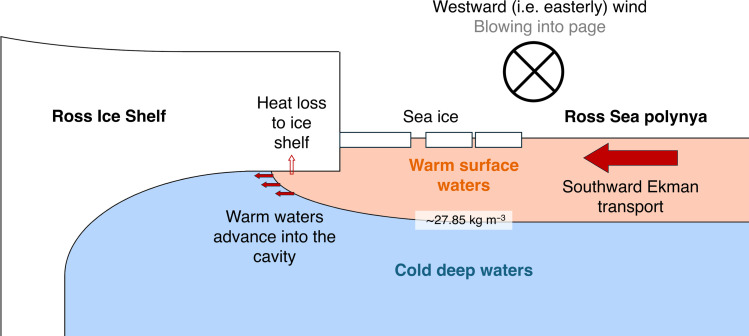
Ekman heat transport into the Ross Ice Shelf cavity. Schematic illustration of surface Ekman heat transport into the cavity of the Ross Ice Shelf.

Given the zonal orientation of the front of the Ross Ice Shelf, an easterly (i.e., westward) wind component will drive a surface Ekman flow toward the ice front and into the cavity (Fig. 4). The potential for this flow to drive basal melting is likely greatest in summer, when the ice-front polynya is open and the austral sun shines on the surface ocean. When the zonal wind component is westerly (i.e., eastward), the surface Ekman flow will be northward and will instead remove water from the cavity. The surface ocean responds to a change in wind stress within a day or two, so even sporadic and transitory wind events could force intrusions even where the prevailing wind is not conducive to the forcing of Ekman inflows.

**Fig. 5.**
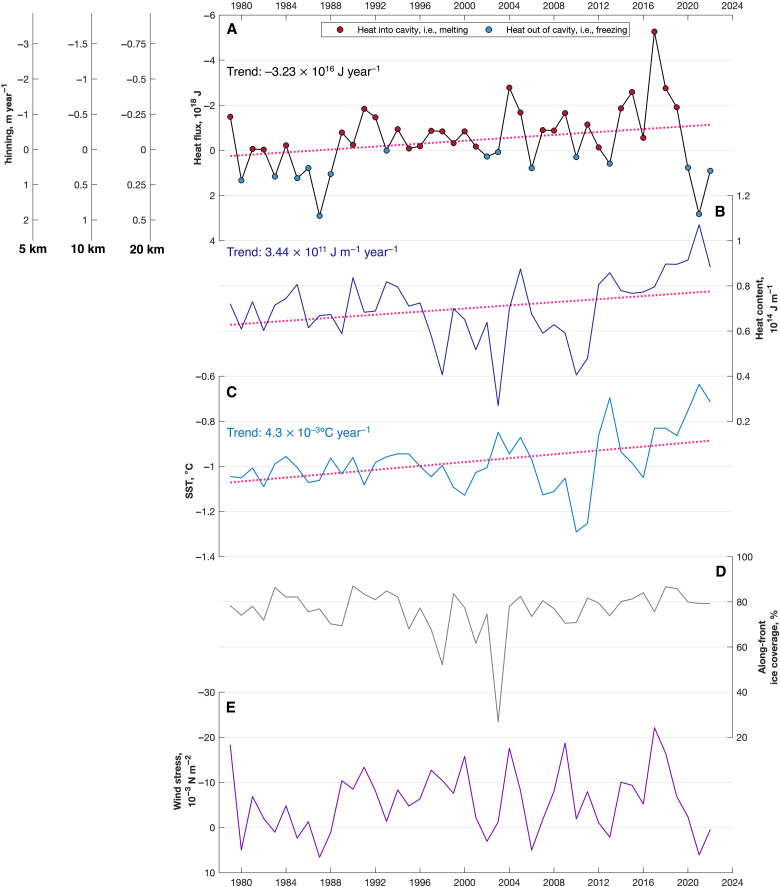
Time series of meridional Ekman heat transport into the Ross Ice Shelf cavity. (**A**) Annually and spatially (i.e., along-front) integrated meridional Ekman heat transport (J). Blue dots indicate heat loss from the cavity; red dots indicate heat gain by the cavity. Assuming that this heat is lost to the overlying ice within 5, 10, or 20 km of the ice front, the three left-hand axes present the same curve as estimates of ice shelf thinning (m year^−1^). Note that the *y* axes in (A) are reversed. (**B**) Annual mean heat content (J m^−1^) of the Ekman layer immediately north of the ice front. (**C**) Annual mean SST (°C) immediately north of the ice front. (**D**) The annual mean percentage of the ice front that is adjacent to open water. (**E**) Annual mean zonal wind stress (N m^−2^) immediately north of the ice front. Note that all variables are defined over open water, i.e., where the sea ice concentration is below 15%. In (A) to (C), the linear trend is indicated by the pink dotted lines.

Integrated over the full width of the Ross Ice Shelf (orange line in Fig. 1), the annual Ekman heat transport across the ice front is generally between ±2 × 10^18^ J (Fig. 5A). If we assume that the ice is the only heat sink for upper-ocean heat within the cavity—i.e., that downward diapycnal mixing of heat out of the Ekman layer is negligible—then we may estimate the basal mass loss as an annual thinning rate; any downward mixing of heat out of the Ekman layer would reduce that available to drive basal melting, so our estimates represent the upper limit of mass loss from the surface Ekman heat transport. Given a range of reasonable distances over which the heat may be absorbed [5 to 20 km; modeling does not suggest that surface water intrudes very far under the Ross Ice Shelf (*15*)], thinning rates that correspond to the Ekman heat transports reported here (Fig. 5A) represent a considerable part of the overall thinning rates immediately south of the ice as derived from through-ice moorings [1.2 ± 0.5 m year^−1^ (*32*)] and on-ice radio echo sounder observations [2 to 3 m year^−1^ (*11*)]. These near-front thinning rates are themselves an order of magnitude greater than the shelf-wide average (*32*, *33*).

Since 1979, there has been a trend toward a greater Ekman heat transport into the Ross Ice Shelf cavity (Fig. 5A; significant at the 90% level, *P* = 0.065): Each year, an additional 3.23 × 10^16^ J of heat energy is advected into the cavity. This trend is associated with an upward trend in Ekman layer heat content (Fig. 5B; 3.44 × 10^11^ J m^−1^ year^−1^; significant at the 90% level, *P* = 0.053) and sea surface temperature (SST; 4.3 × 10^−3^°C year^−1^; Fig. 5C; significant at the 99% level, *P* = 0.005) along the front—i.e., in open waters of the polynya immediately north of the ice front. We note that there is no trend in either annual mean along-front sea ice coverage or annual mean along-front zonal wind stress (Fig. 5, D and E). Interannual variability in the across-front heat flux is not sensitive to annual mean along-front heat content (Fig. 5B; *R*^2^ = 0.01) nor to annual mean sea ice cover (Fig. 5D; *R*^2^ << 0.01). Rather, interannual variability is controlled by annual mean wind stress (Fig. 5E; *R*^2^ = 0.76).

## DISCUSSION

Our observations characterize the hydrography in the uppermost reaches of an ice shelf cavity during a warm surface-water intrusion as it crosses the ice front, as well as demonstrating the sharp hydrographic boundaries that such an intrusion can create. Furthermore, they provide a depth scale and a quantitative description of the boundary layer (Eq. 2). Observations of this boundary layer, particularly ones with a high density of observations in either space or time, are reported only rarely in the literature. For instance, Arzeno *et al.* (*32*) report only five near full-depth temperature and salinity profiles from beneath the Ross Ice Shelf that resolve the under-ice boundary layer and use a long-term single-point observations to estimate melt rate, Nelson *et al.* (*17*) report under-ice dives similar to those presented here but do not discuss the under-ice boundary layer, and the observations presented by Stewart *et al.* (*11*) do not cover the top 8 m of the cavity.

An exponential decrease in temperature toward the ice base has been predicted to occur when eddy diffusivity is constant with depth (*34*, *35*). When forced with heat loss to the ice such that the uppermost point is at the freezing point temperature, the cold anomaly diffuses downward in the water column with time, weakening the temperature gradient (*34*, *35*). The resemblance of the observed mean warm-water temperature profile to this modeled scenario suggests that a depth-independent eddy diffusivity, at least over the upper 5 m, is the most appropriate explanation of near-ice vertical heat transfer in our observations.

Previous modeling works have assumed that the temperature in the centimeter or so immediately beneath the ice is at the in situ freezing temperature (i.e., zero thermal driving) (*34*, *35*), but this is not the case in our observations. When solar-warmed surface water is in contact with the ice, the uppermost observed temperatures remain above the in situ freezing point, and the vertical temperature gradient is thus smaller than has been previously assumed (*34*, *35*). Given the proximity of our observations to the ice front, it may be that the warm water has not been in contact with the ice for long enough for its temperature to fall to the in situ freezing temperature; future modeling work could examine this question. However, a limitation of our work is the potential for part of the uppermost cavity to remain unobserved, the temperature-salinity sensor being part way along a Seaglider sitting at an angle to the ice base. Having no independent observations of ice draft, we must assume that the glider’s uppermost observation on each under-ice profile is at the ice base (i.e., we define this as *z*′ = 0), but we acknowledge that there may be a distance of around 0.1 to 0.15 m between this observation and the true ice base. We note again, however, that the glider’s pitch decreases from ~30° to ~10° within the top 1 m as the still-buoyant glider flattens itself against the ice base (Fig. 3A).

We find that interannual variability in the Ekman heat flux, and in the melting to which it gives rise, is driven by interannual variability in along-front wind stress; the background, multidecadal trend toward greater Ekman heat fluxes into the Ross Ice Shelf cavity is associated with an increase in SST and Ekman layer heat content adjacent to the ice front. It appears reasonable to expect that the magnitude of the Ekman heat flux, and of the melting that it drives, will increase yet further as climate change drives further ocean warming. This trend is a concern in itself, but the proximity of the cavity beneath the Ross Ice Shelf to the in situ freezing temperature heightens the influence of processes that can perturb this state. In West Antarctica, surface-water intrusions into ice shelf cavities might be expected to have a smaller influence on basal melting than the large-scale intrusions of CDW. However, in the Ross Sea, and in other of the cold-water seas around Antarctica in which CDW is not near ubiquitous over the continental shelf, the influence of warm surface-water intrusions on basal melting could be more profound.

This analysis does not capture surface-water intrusions arising from processes other than wind stress; for instance, previous works have identified density-driven flows close to and under the Ross Ice Shelf (*30*–*32*), and density-driven currents have been linked to surface-water intrusions into the cavity (*11*). Nevertheless, given we find that the trend toward greater Ekman heat transport into the cavity is associated with an increase in SST and Ekman layer heat content, this warming trend will likely apply to surface-water intrusions no matter their forcing. A comprehensive analysis of the relative importance of density-driven and wind-driven flows on the transport of heat beneath the Ross Ice Shelf would be an important question for future modeling work.

The correct representation of heat and salt advection directly beneath ice shelves has been identified as necessary to improve the accuracy of modeled melt rates (*36*). Our results provide a benchmark against which model performance may be assessed, giving the mechanistic understanding necessary to determine if processes inside and outside of an ice shelf cavity are being correctly represented. The influence of surface-water intrusions on the upper-cavity boundary layer and on basal melting, alongside the trends and variability in the Ekman dynamics that can drive these intrusions, must be incorporated into climate models, not least given continued uncertainty in the response of Antarctic land–based ice to climate change.

## MATERIALS AND METHODS

### Hydrographic observations

The Seaglider was equipped with a conductivity-temperature sensor and sensors to measure optical backscatter, dissolved oxygen, and chlorophyll fluorescence. Post-deployment, the thermal lag of the conductivity-temperature sensor was corrected (*37*) and the hydrodynamic flight model was optimized (*38*). Salinity observations were visually inspected and erroneous measurements, such as might arise from slow flow past the sensor, were removed. Particulate backscatter was calculated from the volume scattering function (i.e., raw backscatter observations) observed by the glider (*39*, *40*) using a centroid angle of 124° and a wavelength of 700 nm; the wavelength and centroid angle are properties of the instrument. Pressure readings were corrected for the vertical offset between the pressure and conductivity-temperature sensors on the glider, which varies with the glider’s pitch.

### Under-ice temperature and salinity profiles

We consider each conservative temperature and absolute salinity profile as a function of distance-below-ice (Eq. 1), where we must define the depth of the ice base as the depth of the uppermost observation taken on each upcast or downcast. We interpolate distance-below-ice profiles onto a vertical resolution of 0.1 m. We then separate warm-water and cold-water profiles (41 and 29 profiles, respectively) as defined above and subtract the temperature at 5 m (Eq. 1). We fit an exponential function to the mean warm-water relative temperature profile (Eq. 2; Fig. 3B).

We assume that the observed temperature decrease toward the ice base is driven by loss of heat to the overlying ice shelf. Assuming that the mean cold-water profile is representative of a situation without heat loss to the overlying ice shelf, we calculate the heat loss from the warm-water profile, *Q*, according to

$$Q=\int_{-5}^{0}\left({\bar{T}}_{\text{cold}}-{\bar{T}}_{\text{warm}}\right)\;\mathrm{dz}\cdot \rho \cdot c_{p}$$ (3)

where ${\bar{T}}_{\text{cold}}$ and ${\bar{T}}_{\text{warm}}$ are the mean warm-water and cold-water profiles, respectively, *c_p_* = 3986.5 J kg^−1^ K^−1^ is the heat capacity of seawater, and ρ = 1027.8 kg m^−3^ is the density, both given a conservative temperature of −1.75°C and an absolute salinity of 34.7 g kg^−1^.

### Ekman heat transport and ice thinning rates

**Fig. 6.**
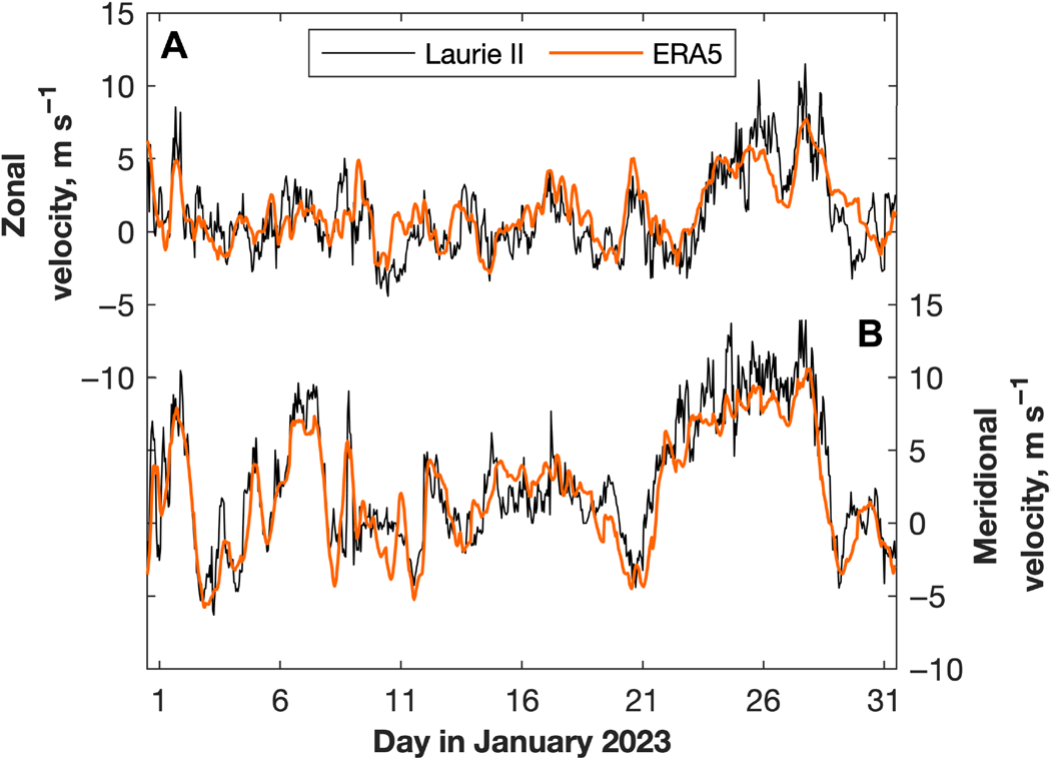
ERA5 and observed wind velocities. (**A**) Zonal and (**B**) meridional wind velocities from the automatic weather station Laurie II (thin black line) and from ERA5 (thick orange line).

We use SST, meridional ocean surface stress (i.e., stress from wind and/or sea ice), and sea ice concentration from the ERA5 reanalysis (*41*) to calculate meridional Ekman mass and heat transport across the front of the Ross Ice shelf between 1979 and 2022 (i.e., since the start of the satellite era). Wind from ERA5 compares favorably to observations from the nearby Laurie II automatic w eather station on the Ross Ice Shelf (Fig. 6). The root mean square difference between ERA5 and the observations in January 2023, the month closest to the glider observations for which a near month-long observational time series is available, is 1.43 m s^−1^ for the zonal velocities and 2.06 m s^−1^ for the meridional velocities. Over the same month, the mean differences (observations minus ERA5) are −0.29 m s^−1^ for the zonal velocities and 0.70 m s^−1^ for the meridional velocities. Both the root mean square differences and mean differences are typical for the random selection of months sampled.

Hourly output is averaged to daily resolution prior to the calculation; output is on a quarter-degree grid in latitude and longitude (~28 by 7 km in the southern Ross Sea). We take output from the southernmost ocean grid points in the Ross Sea—i.e., between 165°E and 160°W. As we are interested in the flux of solar-warmed surface waters into the ice shelf cavity, we isolate the open waters of the polynya (Fig. 1) by masking all points with a sea ice concentration of greater that 15%. The front of the Ross Ice Shelf is approximately zonal, so we assume that cross-frontal volume and heat fluxes are well approximated by meridional Ekman fluxes.

We first calculate meridional Ekman volume transport, *V*_Ek_, according to

$$V_{\text{Ek}}=-\frac{\tau_{u}}{f\rho }$$ (4)

where τ*_u_* is the zonal surface ocean stress, *f* is the Coriolis parameter, and ρ is the density calculated using SST and an assumed sea surface salinity of 34.5 PSU (practical salinity unit). [Sea surface salinity for the southern Ross Sea is taken from the World Ocean Atlas 2018 (*42*)]. We assume that surface temperatures and salinities are representative of temperatures and salinities over the Ekman layer. We then calculate the meridional heat transport, *V*_ht_, associated with *V*_Ek_

$$V_{\text{ht}}=\rho \cdot c_{p}\cdot \left(T_{\text{SST}}-T_{f}\right)\cdot V_{\text{Ek}}$$ (5)

where *T*_SST_ is SST, *T*_f_ = −1.93°C is freezing point temperature given our assumed salinity. We integrate *V*_ht_ over each year and over the width of the ice front (i.e., between 165°E and 160°W) to estimate the heat flux across the ice front. We divide each year’s heat flux by the latent heat of fusion to calculate the mass of ice, *M*_ice_, that would be melted (positive heat flux, i.e., into the cavity) or created (negative heat flux) by that heat flux. This assumes that, within the cavity, the ice shelf is the only source or sink for the heat transported by surface waters. Last, by assuming that *V*_ht_ is absorbed by the ice shelf over a given distance, *L*, from the front—for instance, 10 km—we estimate the annual thinning of the ice sheet, ∆*H*

$$\Delta H=\frac{M_{\text{ice}}}{\rho_{\text{ice}}\mathrm{WL}}$$ (6)

where ρ_ice_ = 918 kg m^−3^ is the density of ice and *W* = 1025 km is the width of the Ross Ice Shelf (i.e., along-front distance).

### Statistical analysis

To assess the goodness of fit between our mean warm-water temperature profile and the exponential fit to that profile (Eq. 2), we consider the coefficient of determination, *R*^2^, between the two sets of temperature values. To assess the statistical significance of the linear trend lines fitted to the time series of Ekman heat transport (Fig. 5A), ice-front Ekman layer heat content (Fig. 5B), and ice-front SST (Fig. 5C), we consider the *P* values of those trend lines—i.e., the probability that such a trend could occur by chance. We again use *R*^2^ to assess the goodness of fit between (i) the heat transport and sea ice and (ii) the heat transport and mean zonal wind time series. The values of all these statistics are cited where relevant in the main text.
