# Supplementary material for: Ross Ice Shelf frontal zone subjected to increasing melting by ocean surface waters
Source: Sci Adv. 2024 Nov 8;10(45):eado6429. doi: 10.1126/sciadv.ado6429 (PMC11546744; doi:10.1126/sciadv.ado6429)
Supplement: Supplementary file 1 — Supplementary Text Figs. S1 to S4 [file sciadv.ado6429_sm.pdf]

Science Advances
AAAS

Supplementary Materials for

Ross Ice Shelf frontal zone subjected to increasing melting by ocean surface waters

Peter M. F. Sheehan and Karen J. Heywood

Corresponding author: Peter M. F. Sheehan, p.sheehan@uea.ac.uk

Sci. Adv. **10**, eado6429 (2024)
DOI: 10.1126/sciadv.ado6429

This PDF file includes:

Supplementary Text
Figs. S1 to S4

Estimating the glider's under-ice trajectory

Given that the glider cannot communicate with the GPS satellite network unless at the surface, we lack a record of the glider's trajectory underneath the ice. We attempted to reconstruct this trajectory using the following methods.

Ice draft

We tried to use the glider-detected draft of the Ross Ice Shelf to constrain the glider's position; maps of ice draft are presented in Figure S1, below. We know from visual observations in the field that the front of the Ross Ice Shelf was some 100 to 200 m from the deployment location of the glider; however, according to BedMachine v3, the front of the Ross Ice Shelf was approximately 11 km from this location (Figure S1). Consequently, we do not feel confident using BedMachine to constrain the glider's position – and BedMachine v3 is the most accurate ice draft and bathymetry product currently available.

Tides

Tidal currents in the region varied between –0.1 and 0.1 m s^{-1} during the glider's under-ice foray (Figure S2; CATS2008 tide model; references and DOI in main paper.) Purely tidal advection would have taken the glider approximately 2 km to the south of its deployment location (Figure S3). However, we do not consider it likely that the glider was advected purely by the tide, as it did not return to its deployment location after a diurnal cycle. Further, purely tidal advection would have taken the glider north of its deployment location at the ice front and thus into open water (Figure S3).

Velocity estimates from our campaign

Unfortunately, we lack direct observations of velocity. Given the helicopter-supported nature of the deployment – it was lowered into the water from the edge of the fast sea ice on 4 December 2022 – we do not have ADCP observations near the deployment site. And the recovery of the glider was over six weeks later (18 January 2023) and 121 km to the north: ship observations from the time are of little help.

We do have the glider's own estimate of its motion through the water: this is optimized post-deployment by the fitting of the hydrodynamic flight model, and is the glider's motion relative to the water – note that it is not distance over ground. This trajectory is plotted in grey on Figure S4. We calculate tidal displacement (Figure S3) and add this to the glider's displacement relative to a stationary water column; this tidally modified glider trajectory is plotted in dark blue on Figure S4. Finally, from the start and end locations of the glider's under-ice foray, we calculate the necessary net background flow (u = 0.045 m s^{-1}, v = –0.028 m s^{-1}). The resulting reconstructed trajectory, comprised of all three components, is plotted in light blue on Figure S4.

Unfortunately, we think that the reconstructed trajectory is likely to be an underestimate of the glider's southward foray: the reconstruction suggests that the glider ventured further north than

the point at which it emerged from the ice. This would have brought the glider into open water earlier than was the case. We also expect that the glider travelled initially more southward than suggested by this reconstructed trajectory, given that it completed many more dives under the ice shelf (to the south) than under the sea ice (to the east).

Order of magnitude estimates

Given that we cannot estimate an under-ice trajectory in which we have confidence, we calculate instead an approximate estimate of the southward distance that the glider traveled beneath the ice. Stewart et al. (ref. 11) report velocities observed by a through-ice mooring between January 2011 and December 2014, averaged between 10 m below the ice base to 30 m above the sea bed. The mooring was 30 km away from the deployment location of our glider, and was 7 km south of the ice front. The background flow observed by this mooring was between approximately 0.01 and 0.04 m s^{-1} (their Figure 2a), and was directed towards the south or southeast. Given these observations of the background current, we estimate that the glider traveled between 1 and 5 km southward under the ice from its deployment location (i.e. implying a "round-trip" distance of 2 to 10 km). Specifically, during an under-ice foray of four days, the maximum southward penetration could be achieved in two days: and in two days, the glider would have traveled:

- 1.7 km at an average speed of 0.01 m s^{-1}
- 3.5 km at an average speed of 0.02 m s^{-1}
- 5.2 km at an average speed of 0.03 m s^{-1}

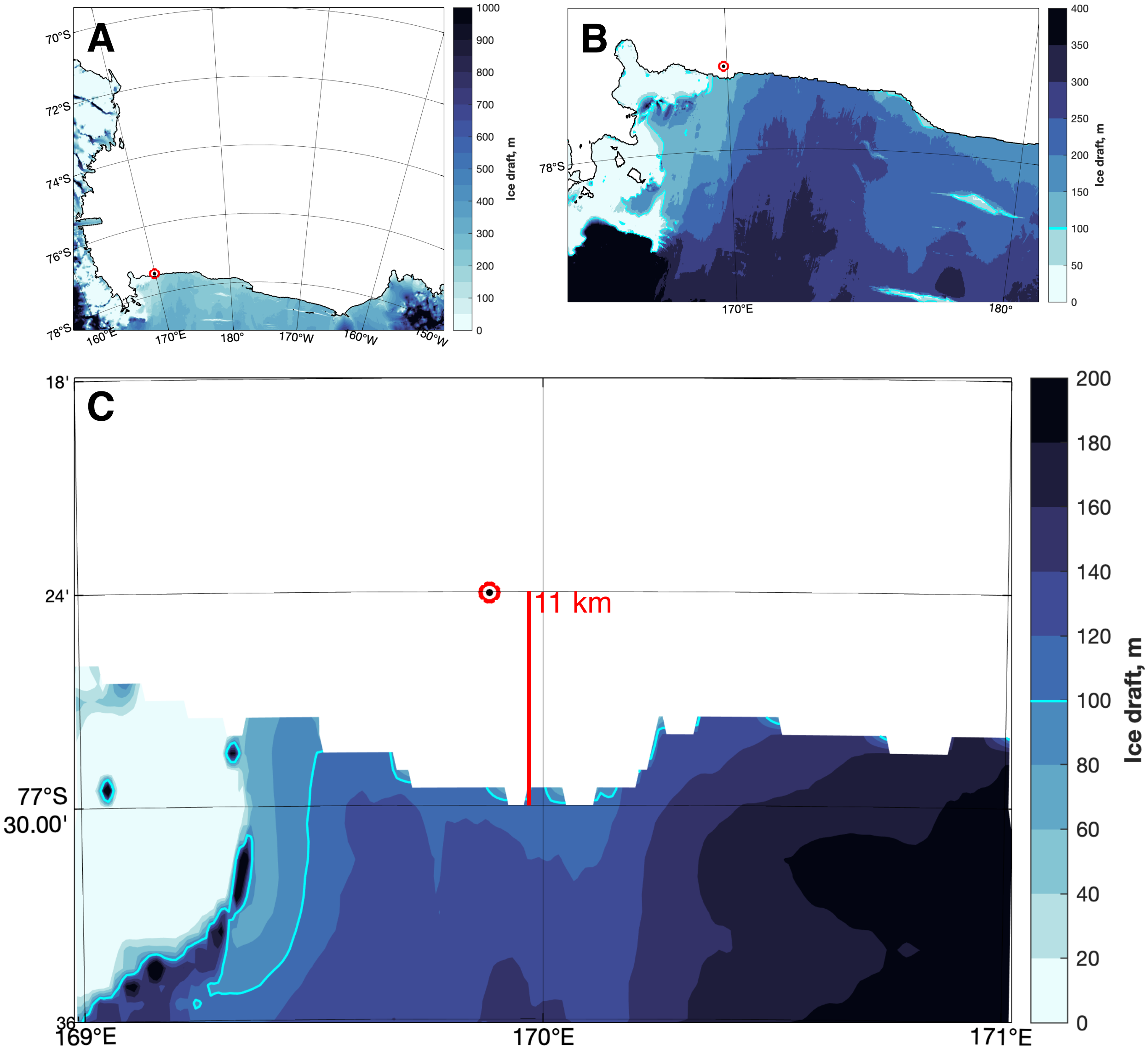

Figure S1. Maps of the draft (m) of the Ross Ice Shelf from BedMachine v3. Note the different color scales on each panel. In panels B and C, the 100 m contour is indicated by the bright blue line. In all panels, the glider's deployment location is indicated by the black dot in the red circle.

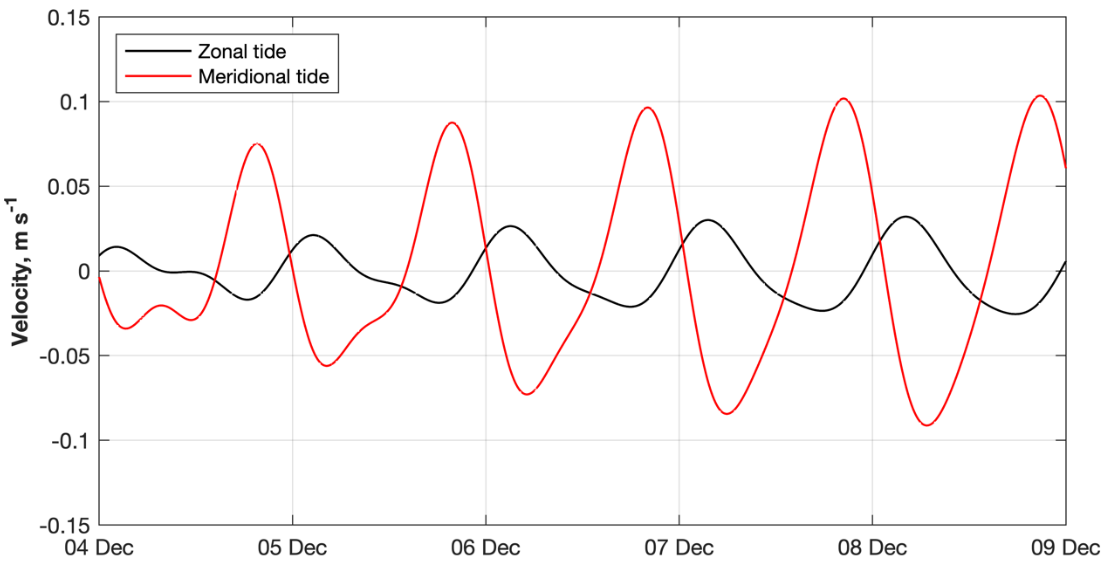

Figure S2. Zonal (black line) and meridional (red line) tidal velocities (m s^{-1}) during the glider's under-ice foray.

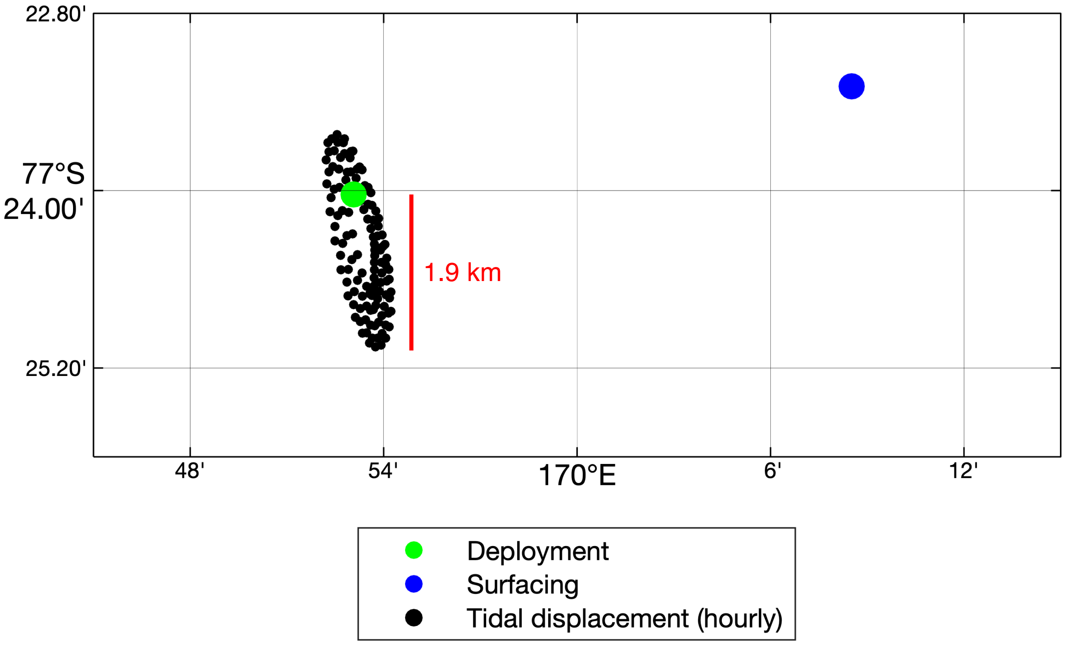

Figure S3. The location of the glider during the period of its under-ice foray, were it advected purely by the tidal currents plotted in Figure S2, above. The location of the glider's deployment and first surfacing are indicated by the green and blue dots respectively.

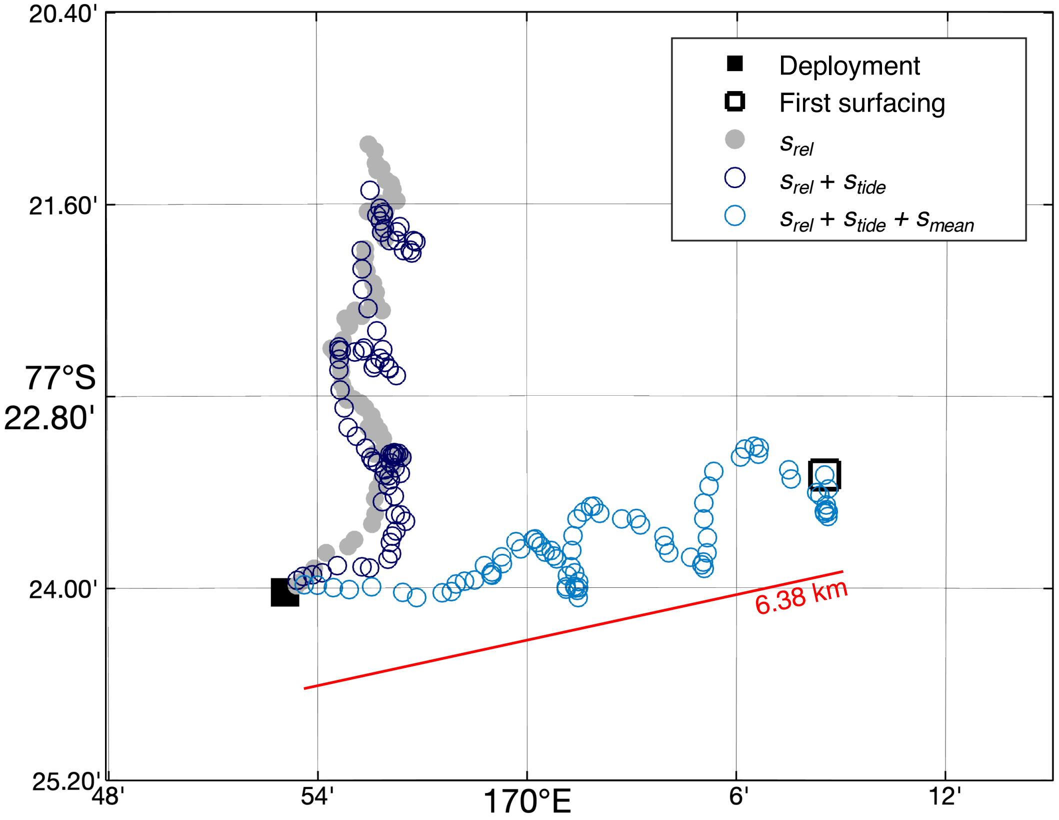

Figure S4. Stages in the reconstruction of SG613's under-ice trajectory. **(1)** In grey, $\boldsymbol{s}_{rel}$: the glider's displacement relative to the water, as estimated during the fitting of the hydrodynamic flight model. **(2)** In dark blue, $\boldsymbol{s}_{rel} + \boldsymbol{s}_{tide}$: displacement relative to the water, modified to include tidal displacement (Figure S3). **(3)** And in light blue, $\boldsymbol{s}_{rel} + \boldsymbol{s}_{tide} + \boldsymbol{s}_{mean}$: displacement relative to the water, modified to include the influence of tides and with an assumed mean flow. The glider's deployment location, and the location at which it first surfaced from under the ice, are shown by the filled and open black squares respectively. The distance between the deployment and first-surfacing locations is 6.38 km.
